# The fluid challenge: Evaluating supportive care in dengue through radiological and clinical markers

**DOI:** 10.6026/973206300221748

**Published:** 2026-03-31

**Authors:** Puja Singh, Neetu Bajaj, Jyoti Namdev, Ranbeer Singh

**Affiliations:** 1 Department of Pathology, Bundelkhand Medical College, Sagar, Madhya Pradesh, India; 2 Department of ENT, Bundelkhand Medical College, Sagar, Madhya Pradesh, India; 3 Department of Pathology, Bundelkhand Medical College, Sagar, Madhya Pradesh, India; 4 Department of Pathology, SMMHMC, Saharanpur, Uttar Pradesh, India

**Keywords:** Dengue, dengue fever, platelet count, blood transfusion

## Abstract

Dengue poses significant health risks and its treatment via platelet transfusions is costly and impractical in resource-limited settings. Therefore, it is of interest to explore fluid resuscitation as a viable alternative to support hemodynamic stability and enhance platelet recovery. Strong correlations were noted between radiological findings and laboratory parameters. Remarkably, all patients, even the severely affected, recovered without transfusions, with prognosis influenced by factors such as liver function and capillary leakage. Thus, we show that platelet count does not solely indicate disease severity and effective fluid resuscitation can manage dengue effectively without the need for blood transfusions.

## Background:

Humans contract dengue through bites from Andes mosquitoes. It poses a significant public health challenge in tropical and sub-tropical regions globally [1]. In severe dengue cases, such as dengue hemorrhagic fever (DHF) and dengue shock syndrome (DSS), it can lead to thrombocytopenia and coagulopathy. Platelet transfusions have traditionally managed thrombocytopenia [2]. However, there are associated risks, such as transfusion reactions [3, 4]. In addition, costly procedures and the short lifespan of platelets make transfusion practically nonviable, especially in resource-deficit regions [5, 6]. Recent clinical research advances have highlighted managing severe dengue cases through administering intravenous fluids and colloid solutions such as albumin [7, 8]. This approach focuses on maintaining hemodynamic stability by fostering endogenous platelet production [8, 9- 10]. Adequate fluid resuscitation can mitigate the severity of bleeding manifestations and enhance the patient's hemostatic profile [11, 12]. Therefore, it is of interest to establish evidence-based practices to ensure optimal patient care for dengue complications while prioritizing safety and efficacy.

## Methodology:

This prospective observational study was conducted at a tertiary care hospital in North India between August and October 2023 to evaluate dengue patient management. The required sample size was calculated as 117, for 95% confidence level (Zα/2 = 1.96), population proportion (p = 0.5) and a 5% margin of error. Eligible participants included patients above 18 years of age with a confirmed positive Dengue ELISA test that provided written informed consent. Patients were excluded if they had pre-existing hematological disorders, chronic liver or kidney disease, malignant tumors, hyperthyroidism, malnutrition, dengue-related hemorrhagic complaints, pregnancy, or if they declined consent. Ethical approval was obtained from the Institutional Ethical Committee (IEC 66/08/02/2024) and informed consent was secured from all participants. Upon presentation, detailed clinical history and symptoms such as fever, rash, bleeding tendencies, abdominal pain and dengue warning signs (persistent vomiting, fluid accumulation, lethargy) were recorded. Vital parameters including blood pressure, heart rate and temperature were documented. All suspected cases underwent ELISA testing for confirmation. Once confirmed, patients were advised hematological, biochemical and radiological investigations and were admitted either to the general ward or ICU depending on clinical severity.

Patients received fluid resuscitation and supportive management as per hospital guidelines. Intravenous fluids were administered in both ICU and ward settings, including albumin, Ringer lactate, normal saline, 5% and 25% dextrose, colloids and antibiotics as required. ICU patients were continuously monitored for vital signs and critical parameters and shifted to the general ward upon improvement. Patients in the ward remained under observation until key parameters normalized or showed significant recovery, after which they were discharged with advice for follow-up after one week. A patient was classified as being in a critical state if one or more of the following criteria were met: platelet count <10,000; total leukocyte counts between 1500-2000; SGOT/SGPT >300 IU/L; serum bilirubin >2 mg/dL; serum creatinine >1.1 mg/dL; pleural effusion or pneumonitic patches on chest X-ray; or edema on abdominal ultrasound. Clinical judgment was also considered in determining severity. Laboratory investigations included hematological tests (platelet count, hematocrit, hemoglobin), biochemical tests (ALT, AST, renal function tests, electrolytes) and coagulation profile (PT, aPTT, INR). Radiological investigations such as chest X-ray and abdominal ultrasound were performed on a case-by-case basis to detect pleural effusion, ascites, or organomegaly. Hypertension and diabetes were considered major confounders. Diabetes was categorized using HbA1c values into nondiabetic (4.0-5.6%), prediabetic (5.7-6.4%) and diabetic (≥6.4%). Hypertension was classified based on systolic and diastolic blood pressure into optimal, normal, high normal, Grade 1, Grade 2, Grade 3 and isolated systolic hypertension. Data on demographic, clinical, laboratory and radiological parameters were recorded in Microsoft Excel and anonymized for confidentiality. Descriptive statistics were used to analyze quantitative variables, expressed as frequencies and percentages. Statistical significance was determined using p-values (<0.05 considered significant). Correlation between laboratory and radiological findings was assessed using Cramer's V for categorical variables. Statistical analyses were performed using Python (v3.9) with NumPy (v1.24), Pandas (v1.3.5) and Seaborn (v0.11.2).

## Results:

The demographic distribution of the dengue patients is presented in Figure 1 (see PDF). The highest number of patients, 136 (45.33%), was found for the age group 21-30, whereas the lowest number, 12(04.00%), for > 60 years. The figure suggests that the disease disproportionately affects younger age groups. Men were more impacted than women. The most common clinical symptoms are fever, body aches and abdominal pain (Table 1 - see PDF). These were reported by all the patients. The most common sign is abdominal tenderness reported by all 300 (100%) patients. It is followed by hepatomegaly in 290 (96.67%) and hypotension (blood pressure less than 70-80 mm Hg systolic) has been observed in 205 (68.33%) patients. The most common hematological test finding was thrombocytopenia (Table 2 - see PDF), with the commonest range of 10,000-20,000 counts/cm. The next finding was leukopenia in 204 (68%) cases. Of these, TLC ranges for 106 (35.33%) were 2000-3000/cm and 52 (17.33%) were 1500-2000/cm, respectively. The most common biochemistry test was serum transaminase (SGPT & SGOT). All 274 (91.33%) cases had raised levels. 169 (56.33%) cases had serum bilirubin tests and all patients had elevated values. All 300 (100.00%) cases had their serum creatinine tested and 77 (25.66%) showed an increase. Data for both hematological and biochemistry tests were statistically significant. 58 patients underwent abdominal ultrasonography (Table 3- see PDF), while 42 patients underwent a chest X-ray. For USG, Hepatomegaly was the most common diagnosis (58 cases), followed by thickening of the gall bladder wall with oedema (52 cases). The most common finding in X-ray chest was unilateral pleural effusion (17 cases), followed by minimum bilateral pleural effusion (10 cases). Findings for both abdominal USGs and chest X-rays were statistically significant. Confounds, hypertension and diabetes, are presented in (Table 4 - see PDF). A total of 25 patients were admitted to the ICU at the time of presentation. About 66.7% of patients either had optimal or normal blood pressure. 19 patients were diabetic. The correlation between laboratory diagnoses and X-ray diagnoses was performed using Cramer's V statistics (Table 5 - see PDF). Serum creatinine and platelet count had the strongest correlation with X-ray diagnosis. We obtained test statistics of 1.00 and 0.8157, respectively. TLC showed the lowest correlation, with test statistics equal to 0.4912. The correlation between laboratory diagnoses and abdominal USG was performed using Cramer's V statistics (Table 6 - see PDF). SGOT & SGPT had the strongest correlation with abdominal USG, with test statistics of 0.6588. TLC showed the lowest correlation, with test statistics equal to 0.3970.

## Discussion:

The WHO classifies dengue as a neglected tropical disease due to limited funding, political commitment and global collaboration [7]. Although blood transfusion is commonly used in severe dengue cases, it remains difficult in resource-limited settings where the disease is widespread. This study evaluates the feasibility of managing dengue primarily through fluid resuscitation while avoiding blood transfusion and also examines correlations between laboratory and radiological findings. This study found a higher incidence of dengue among males (79.67%) compared to females (20.33%), consistent with findings by Loi *et al.* [8]. However, Tayal *et al.* [9] reported nearly equal gender distribution, while Vidanapathirana [10] observed more female cases. The incidence of dengue decreased with increasing age, possibly because males are more involved in outdoor activities due to occupational responsibilities, increasing their exposure to mosquitoes. Similar observations were reported by Loi *et al.* [8] and Keshav *et al.* [11] most cases occurred between August and October, coinciding with the post-monsoon mosquito breeding season in the region. Keshav *et al.* [11] supported this seasonal trend. Recognizing this clustering can help hospitals strengthen preparedness and assist local authorities in implementing more effective vector control strategies. The key morbidity indicators identified in this study were shock, leukopenia, gall bladder wall edema, peritoneal fluid, pleural effusion and severe hepatitis (SGPT >300). Unlike previous studies that evaluated predictors individually, this study assessed them collectively. Zafar *et al.* highlighted shock, coma and convulsions as significant mortality predictors [12]. Consistent with Hasibuan [13] and Vidanapathirana [10], shock was recognized as a major marker of disease severity. Although 33.33% of patients developed shock, none experienced bleeding diathesis or death. While Rahman *et al.* [14] linked spontaneous bleeding with higher mortality, no such association was observed here. Severe thrombocytopenia was common, with some patients having platelet counts below 10,000/cmm and most between 10,000-20,000/cmm; however, all recovered without platelet transfusion through effective fluid resuscitation. Other researchers, including Vidyashree *et al.* [15] and Trieu *et al.* [4], concluded that platelet count alone does not determine prognosis and platelet transfusion may not reduce severe bleeding, warranting cautious use. In this study, hepatitis with high transaminase levels and leucopenia were reported in 274 (91.33%) and 204 (68%) cases, respectively. All the patients recovered from hepatitis with treatment and TLC and platelet count returned to normal. Salahuddin *et al.* observed that dengue infection caused leukopenia [16]. In this study, 77 (25.66%) patients had slightly elevated serum creatinine levels. However, as patients recovered from shock, leukopenia and hepatitis, creatinine levels returned to normal. Thus, this finding did not significantly affect the patient's prognosis. This study observed that dengue fever manifested as an acute febrile illness with a wide range of constitutional symptoms and no specific symptoms. In this study, severe disease was associated with an increase in transaminase. Severe liver disease caused thrombocytopenia. Htun [17] and Mwanyika *et al.* [18] described similar findings and concluded that hepatic dysfunction in dengue was the major determinant of the prognosis of the illness. Two confounders, diabetes and hypertension, were studied in this study. Diabetes was reported for 6.33% of all the patients in this study and 16% among ICU patients. Wang *et al.* reported 4.59% of diabetes in all patients and 8.82% in severe cases [6]. Guingané *et al.* reported diabetes in 2.15% of the patients [19]. Hypertension was reported in 4.67% of all the cases in this study and 20% among ICU patients. Wang *et al.* reported 12.56% of hypertension in all patients and 32.35% in severe cases [6]. Guingané *et al.* reported hypertension in 6.45% of the patients [19]. Data for other confounders, such as chronic lung disease and cerebrovascular disease were not captured in this study. A strong correlation was observed between laboratory parameters and radiological findings. Creatinine showed a perfect correlation (r=1) with X-ray findings, while platelet count demonstrated a strong correlation (r=0.8157). SGOT and SGPT correlated well with abdominal ultrasonography (USG), though overall X-ray findings showed better correlation with laboratory results than USG. Previous studies have also explored associations between platelet count, albumin and dengue outcomes. Despite several severe thrombocytopenia cases, no deaths or bleeding were recorded. All patients were managed conservatively with routine ward care, including fluids, antibiotics, multivitamins, steroids, diuretics and supportive treatment for hepatitis and effusions. Other studies, Sangkaew *et al.* [20] and Wang *et al.* [6] also observed similar findings. We observed that prompt early clinical diagnosis and hydration control are key to preventing adverse effects, as all the patients recovered without any further complications.

## Limitations and future scope of work:

To generalize a recommendation for platelet transfusion, wider Pan-India tertiary center reports are required. Case-control observation studies comparing outcomes from fluid resuscitation and blood transfusion can help accurately demonstrate the comparative benefits. The study did not cover any cases of haemorrhage and did not explore the role of comorbidities such as chronic lung disease and cerebrovascular disease.

## Conclusion:

We show factors such as abnormal liver functions, capillary leakage causing effusions, shock and leucopenia notably affect dengue prognosis, while platelet count alone does not determine severity or treatment options. X-ray results correlate more closely with laboratory findings than abdominal ultrasounds. Effective routine care with fluid resuscitation can manage dengue patients, especially in resource-limited areas, but further research is needed concerning patients with very low platelet counts and hemorrhagic symptoms.
